# *In vivo* serotonin 1A receptor hippocampal binding potential in depression and reported childhood adversity

**DOI:** 10.1192/j.eurpsy.2023.4

**Published:** 2023-01-24

**Authors:** Elizabeth A. Bartlett, Ashley A. Yttredahl, Maura Boldrini, Andrea E. Tyrer, Kathryn R. Hill, Mala R. Ananth, Matthew S. Milak, Maria A. Oquendo, J. John Mann, Christine DeLorenzo, Ramin V. Parsey

**Affiliations:** 1 Department of Psychiatry, Columbia University Irving Medical Center, New York, New York 10032, USA; 2 Molecular Imaging and Neuropathology Division, New York State Psychiatric Institute, New York, New York 10032, USA; 3 Department of Psychiatry, Stony Brook Medicine, Stony Brook, NY 11794, USA; 4 Clinical Genetics Research Program, Centre for Addiction and Mental Health, University of Toronto, Toronto, Ontario M5S, Canada; 5 National Institute of Neurological Disorders and Stroke, National Institute of Health, Bethesda, Maryland 20892, USA; 6 Department of Psychiatry, Perelman School of Medicine, University of Pennsylvania, Philadelphia, Pennsylvania 19104, USA; 7 Department of Radiology, Columbia University, New York, New York 10027, USA; 8 Department of Biomedical Engineering, Stony Brook University, Stony Brook, New York 11794, USA; 9 Department of Radiology, Stony Brook University, Stony Brook, New York 11794, USA

**Keywords:** Serotonin 1A receptor, positron emission tomography, childhood adversity, depression

## Abstract

**Background:**

Reported childhood adversity (CA) is associated with development of depression in adulthood and predicts a more severe course of illness. Although elevated serotonin 1A receptor (5-HT_1A_R) binding potential, especially in the raphe nuclei, has been shown to be a trait associated with major depression, we did not replicate this finding in an independent sample using the partial agonist positron emission tomography tracer [^11^C]CUMI-101. Evidence suggests that CA can induce long-lasting changes in expression of 5-HT_1A_R, and thus, a history of CA may explain the disparate findings.

**Methods:**

Following up on our initial report, 28 unmedicated participants in a current depressive episode (bipolar *n* = 16, unipolar *n* = 12) and 19 non-depressed healthy volunteers (HVs) underwent [^11^C]CUMI-101 imaging to quantify 5-HT_1A_R binding potential. Participants in a depressive episode were stratified into mild/moderate and severe CA groups via the Childhood Trauma Questionnaire. We hypothesized higher hippocampal and raphe nuclei 5-HT_1A_R with severe CA compared with mild/moderate CA and HVs.

**Results:**

There was a group-by-region effect (*p* = 0.011) when considering HV, depressive episode mild/moderate CA, and depressive episode severe CA groups, driven by significantly higher hippocampal 5-HT_1A_R binding potential in participants in a depressive episode with severe CA relative to HVs (*p* = 0.019). Contrary to our hypothesis, no significant binding potential differences were detected in the raphe nuclei (*p* *-value* *s* > 0.05).

**Conclusions:**

With replication in larger samples, elevated hippocampal 5-HT_1A_R binding potential may serve as a promising biomarker through which to investigate the neurobiological link between CA and depression.

## Introduction

Bipolar depression (BD) and major depressive disorder (MDD) are among the leading causes of disability worldwide [1]. Reported childhood adversity (CA) is a predictor of lifetime risk for MDD [2] and is associated with poorer prognosis in both MDD and BD [3, 4]. Aberrations of the serotonergic system, strongly implicated in the diathesis of depression [5], may be a result of CA and may moderate the relationship between adversity and onset and progression of depressive symptomatology [6, 7].

More than other types of negative life events, CA is associated with depressive disorders [2]. Reported CA has a high prevalence in adults with mood disorders [8], and episodes of adversity have a cumulative effect on the risk for depression in adulthood [9]. CA plays a large role in clinical outcomes as well [10], predicting an earlier age of onset of MDD and BD, more persistent and frequent depressive episodes, and increased risk for suicide attempts [3, 4].

One possible mechanism by which CA influences depression and its severity is through the serotonin system, including increases in serotonin 1A receptor (5-HT_1A_R) density [10–12]. Alterations in positron emission tomography (PET)-measured 5-HT_1A_R binding potential can predict antidepressant treatment efficacy in both BD and MDD [13, 14]. Animal research has shown altered 5-HT_1A_R expression following early life adversity [15, 16, 17, 18]. Glucocorticoid-induced down-regulation of the 5-HT_1A_R occurs across the lifespan, likely an adaptive response to stress [19]. However, evidence suggests that there is a critical developmental window early in life during which exposure to stress can induce more enduring changes in 5-HT functioning [11, 12, 20–22]. In rodents exposed to early life stress, there have been reports of both increases in 5-HT_1A_R density in the hippocampus [18] and attenuated 5-HT_1A_R mRNA expression [21, 23] and 5-HT_1A_R binding [24]. Nonhuman primates with early life stress had lower 5-HT_1A_R density [25] and lower 5-HT_1A_R binding [15]. Rodents exposed to early life stress have also been shown to develop anxious and depressive-like phenotypes in adulthood with increased hippocampal 5-HT_1A_R density [20]. Differing models of early life stress in rodent and nonhuman primate work (e.g., time of maternal deprivation and animal age) may contribute to the mixed findings.

In humans, postmortem work reports higher 5-HT_1A_R density in suicide decedents with a history of CA compared to healthy volunteers (HVs), suicide decedents without a history of adversity, and nonsuicide decedents with a history of adversity [26]. Critically, CA may reduce the efficacy of pharmaceuticals that target the serotonin system [16] via upregulation of the 5-HT_1A_R in the hippocampus [27] and might explain why both higher 5-HT_1A_R binding [28] and a history of CA [4] predict antidepressant treatment resistance.

Studies using PET to quantify 5-HT_1A_R binding potential in MDD and BD have reported different results, largely due to differences in quantification methods [29, 30]. Studies reporting binding potential with reference to the free plasma concentration (BP_F_), which has been the standard in our group, have most commonly found elevated 5-HT_1A_R binding in currently depressed and euthymic patients with MDD and BD relative to HVs [13, 28–34]. Conversely, many studies reporting alternate binding potentials have found lower 5-HT_1A_R binding in depression [35–38]. When we considered the alternate binding potential, BP_ND_, which is in reference to the nondisplaceable radioligand in tissue [39], we reconciled these discrepant findings and found lower 5-HT_1A_R binding in not recently medicated participants with MDD relative to HVs [29]. However, due to the group differences found in reference region uptake [29], we maintain the use of BP_F_ as our standard outcome measure for 5-HT_1A_R quantification. With this, in Ananth et al. [40], although we found that 5-HT_1A_R BP_F_ predicted clinical response to lithium treatment for BD, there were no differences between HVs and participants with BD. The analyses reported herein were conducted using an overlapping dataset to Ananth et al. [40] as a follow-up to evaluate whether CA could explain part of the unexpected null result.

We examined the relationship between CA and 5-HT_1A_R binding potential, an index of 5-HT_1A_R density (assessed with the PET tracer carbon 11–labeled [O-methyl-(11)C]2-(4-(4-(2-methoxyphenyl)piperazin-1-yl)butyl)-4-methyl-1,2,4-triazine-3,5(2H,4H)dione [^11^C]CUMI-101) in a transdiagnostic sample of unmedicated participants in a current depressive episode and HVs. We assessed 5-HT_1A_R binding in the hippocampus because of the sensitivity of this region to early life stress [15, 19, 20, 41, 42]. In addition, we assessed binding in the raphe nuclei because it is the primary site of serotonin synthesis [43, 44]. Further, 5-HT_1A_Rs in this region are autoreceptors that regulate serotonin neuron firing and release and may mediate the antidepressant action of selective serotonin reuptake inhibitors for depression [14]. We hypothesized that differences in 5-HT_1A_R binding potential between participants in a depressive episode and HVs would be driven by CA, such that participants reporting severe CA would also have the highest 5-HT_1A_R binding potential.

## Materials and Methods

### Participants

Twenty-eight participants in a current depressive episode (BD: *n* = 16, MDD: *n* = 12), and 19 non-depressed HVs were included in this analysis and overlap with participants from two other previously reported studies [40, 45]. All bipolar participants from Ananth et al. [40] were used here, except the four participants missing Childhood Trauma Questionnaire (CTQ) data from that study. The hypotheses regarding CA were secondary to the main grant (National Institutes of Mental Health R01MH090276). Data could be made available by request to Dr. Ramin V Parsey. Study procedures were approved by Institutional Review Boards at Brookhaven National Laboratory (BNL), Yale University Medical Center (Yale), Stony Brook University (SBU), and Columbia University Irving Medical Center (CUIMC), and written informed consent was obtained from all participants. Participants were anonymized after intake and assigned unique study identification numbers.

Patients were aged 18–70 years, in a current major depressive episode (scores ≥15 on the 17-item Hamilton Depression Rating Scale (HDRS-17) [46]), and free of psychotropic medications for at least 3 weeks prior to PET scans (except benzodiazepines for at least 24 hours, fluoxetine for at least 6 weeks, and serotonin-depleting drugs for at least 3 months prior to PET scans). Participants were diagnosed with either BD or MDD but were free of any other major psychiatric disorders (not including eating disorders or phobias) as assessed using the Structured Clinical Interview for DSM-IV [47]. HVs had no lifetime history of Axis I disorders (except one HV had a history of adjustment disorder 20 years prior), and no first-degree relatives with a history of major depression (for participants under the median age plus one quartile for depression onset: 44 years), schizophrenia, suicide attempt, or ≥2 first-degree relatives with a history of substance dependence (for participants under 27 years old). All participants were free of recent alcohol or substance dependence (except for cannabis) for the past 6 months, current substance abuse for the past 2 months, intravenous drug use in the past 5 years, and use of ecstasy more than 15 times in the past 10 years or any use in the month prior to scans.

Participants completed the HDRS-17 and Hamilton Anxiety Scale (HAM-A) [48]. CA was assessed using the CTQ [49], and the combined sample of currently depressed participants with MDD and BD was divided into those who reported severe adversity (*n* = 21, comprising 10 MDD participants and 11 BD participants) or no, low, or moderate adversity (*n* = 7, comprising 2 MDD participants and 5 BD participants) based on score classifications in the CTQ manual (see Supplementary Table S1) [49]. Two patients reported moderate adversity, four patients reported low adversity, and one patient reported no adversity; therefore, the no, low, or moderate category will be referred to as “mild/moderate CA” herein. Of the HVs, six patients reported low CA and 13 reported no CA. The CTQ was on average 0.4 ± 59.2 days before the PET scan (max 154 days before, max 227 days after). We did not require close proximity of the CTQ to the PET scan given excellent CTQ stability despite changes in psychopathology levels [50].

### Scanning procedure

#### Image acquisition and analysis

[^11^C]CUMI-101 PET imaging and analysis were performed as previously described [45]. All participants underwent a 120-minute [^11^C]CUMI-101 PET scan, acquired on either an ECAT EXACT HR+ (Yale *n* = 42 and BNL *n* = 4) or ECAT HR+ (CUIMC *n* = 1) (Siemens CTI Molecular Imaging, Knoxville, TN, USA) [40, 45]. Following a 10-minute transmission scan, [^11^C]CUMI-101 was injected as an intravenous bolus (injected dose = 14.65 ± 4.57 mCi; specific activity = 5.20 ± 3.97 mCi/nmol). Automated arterial blood sampling was performed for the first 7 minutes of PET acquisition, with manual sampling thereafter. As previously described, the full arterial sampling data were used to generate a metabolite-corrected input function [45]. In the 13 cases without full arterial sampling data, a simultaneous estimation algorithm validated for [^11^C]CUMI-101 was used to compute the metabolite-corrected input function with the solution constrained by a single venous or arterial blood sample [51–53].

Motion correction was performed via frame-by-frame rigid body registration to a reference frame. The mean of the motion-corrected frames was then co-registered to the participant’s MRI [45]. The *a priori* hippocampus and raphe nuclei regions were delineated as previously described [45, 54, 55] (raphe nuclei mask available at https://renaissance.stonybrookmedicine.edu/psychiatry/research/cubit/data). These delineations were used to create regional time activity curves. Exploratory volumetric analyses were performed using Freesurfer 5.3 (http://surfer.nmr.mgh.harvard.edu/) to automatically segment the whole hippocampus and its subfields [56], following a visual inspection of intermediate preprocessing steps [57], to ensure BP_F_ differences were not influenced by volume differences between groups.

Likelihood estimation in graphical analysis (LEGA) was used to obtain the optimal [^11^C]CUMI-101 outcome measure BP_F_ (= B_avail_ (concentration of available receptors) / K_D_ (radiotracer equilibrium dissociation constant), that is, the ratio of the concentration of specifically bound [^11^C]CUMI-101 in tissue to the concentration of free [^11^C]CUMI-101 in blood plasma at equilibrium) from the hippocampus and raphe nuclei time activity curves [39, 58]. BP_F_ is expressed in units of mL/cm^3^, that is, if BP_F_ = 5 mL/cm^3^, then 5 mL of plasma would be required to account for [^11^C]CUMI-101 in 1 cm^3^ of brain tissue [39]. The plasma-free fraction (f_P_) across participants was 37% ± 7%. Standard errors (SE) were computed via resampled residuals bootstrapping of the LEGA fits [59]. A better fit of the time activity curve corresponds to lower SE and therefore heavier weighting of that participant’s 5-HT_1A_R BP_F_ data in the linear mixed-effects (LME) models.

### Statistical analyses

Differences in age, HDRS-17, and Beck’s Depression Inventory (BDI) [60] between the three groups (HV, participants in a depressive episode with mild/moderate CA, participants in a depressive episode with severe CA) were analyzed using a one-way analysis of variance (ANOVA), and significant effects were followed up with independent samples *t*-tests. Differences in HAM-A and CTQ between the mild/moderate and severe CA depressive episode groups and in HDRS-17, BDI, and age between HVs and participants in a depressive episode were assessed using independent samples *t*-tests. Pearson’s chi-squared test was used to assess group differences in sex distributions. All demographic analyses were conducted in R, version 3.5.3 (R Core Team).

Log-transformed 5-HT_1A_R BP_F_ data (used to stabilize between-region variances) were checked for normality and the absence of outliers. The impact of CA on 5-HT_1A_R BP_F_ was analyzed with a LME model. Log-transformed BP_F_ was the model outcome with fixed effects of brain region (raphe nuclei and hippocampus), group (HV, mild/moderate CA, severe CA), and sex, and participant fit as a random effect. The interaction effect of group-by-region was also fit. BP_F_ values were weighted using the individual SEs, as 1/SE^2^. Reported percent differences between groups were computed using means weighted according to this method. Both within- and between-participant covariances were modeled using unstructured variance and random intercepts, and residual maximum likelihood was used for model fitting. A second, two-group LME was also fit comparing HVs to the full sample of all participants currently in a depressive episode (not splitting by CA status, with BD and MDD combined). Convergence criteria were met for the models reported. These analyses were performed using SAS 9.4 (SAS Institute, Inc., Cary, NC, USA).

Site was excluded from both primary and secondary analyses as a fixed factor due to group imbalance (all patient scans were acquired at Yale). However, sensitivity analyses were conducted by repeating the LMEs for the subset of participants who underwent their PET scans at Yale (*n* = 42) (see Supplementary Materials and Supplementary Figure S1).

## Results

Participants in a depressive episode and HVs did not significantly differ in age or sex (Table 1), and no significant group differences were present in these variables when participants were grouped by CA severity (depressive episode with mild/moderate CA [*n* = 7], depressive episode with severe CA [*n* = 21], and HVs [*n* = 19]; Table 2). Injected dose, injected mass, specific activity, and mean free fraction in blood plasma (f_P_) also did not differ significantly between groups (see Supplementary Table S2). No significant differences were observed between depressive episode groups on other clinical measures including ratings of anxiety, depression, or suicidality, except for CTQ scores, as expected (Table 2).

**Table 1.** Clinical and demographic characteristics of HVs and participants in a depressive episode.

	HVs (*n* = 19)	Participants in a depressive episode (*n* = 28)	Statistical comparison
Age, mean (SD)	40.6 (13.4)	33.0 (13.1)	*t*_38_ = 1.94 (*p* = 0.06)^a^
Female: male ratio	9F:10M	13F:15M	Χ^2^_(1)_ = 0.004 (*p* = 0.94)
Childhood Trauma Questionnaire (CTQ) (SD)	28.9 (4.71)	59.0 (17.0)	*t*_33_ = −8.89 (*p* < 0.001)^a^
Hamilton Anxiety Scale (HAM-A) (SD)	N/A^c^	20.5 (6.29)^b^	N/A^c^
Hamilton Depression Rating Scale (HDRS-17) (SD)	1.89 (2.14)	20.5 (3.51)^b^	*t*_41_ = −21.8 (*p* < 0.001)^a^

Abbreviations: HV, healthy volunteer; SD, standard deviation.

a Welch’s independent sample *t*-test for unequal variances.

b Data missing for two participants.

c HAM-A for HVs not available.

**Table 2.** Demographic and clinical characteristics of HVs, participants in a depressive episode with mild-to-moderate childhood adversity (CA), and participants in a depressive episode with severe CA.

	HVs (*n* = 19)	Participants in a depressive episode with mild-to-moderate CA (*n* = 7)	Participants in a depressive episode with severe CA (*n* = 21)	Statistical comparison
Age, mean (SD)	40.6 (13.4)	30.3 (13.1)	33.9 (13.3)	F_2,44_ = 2.07 (*p* = 0.14)
Female: male ratio	9F:10M	2F:5 M	11F:10 M	Χ^2^_(2)_ = 1.20 (*p* = 0.55)
Childhood Trauma Questionnaire (CTQ) (SD)	28.9 (4.71)	37.7 (8.28)	66.1 (12.5)	F_2,44_ = 81.0 (*p* < 0.001)
Bipolar depression (BD):major depressive disorder (MDD) ratio	N/A	5BD: 2MDD	11BD: 10MDD	Χ^2^_(1)_ = 0.19 (*p* = 0.66)
Hamilton Anxiety Scale (HAM-A) (SD)	N/A^d^	18.9 (8.28)	21.1 (5.79)^c^	*t*_9_ = 0.68 (*p* = 0.51)^a^ ^,^^b^
Hamilton Depression Rating Scale (HDRS-17) (SD)	1.89 (2.14)	18.5 (3.51)^e^	21.2 (3.36)^e^	F_2,41_ = 216.2 (*p* < 0.001) *t*_8_ = 1.60^a^ ^,^^b^ (*p* = 0.14)

Abbreviations: HV, healthy volunteer; SD, standard deviation.

a Welch’s independent sample *t*-test for unequal variances.

b Statistical comparison between participants in a depressive episode groups.

c Data missing for two participants.

d HAM-A for HVs not available.

e Data missing for one participant.

### A priori analyses

**Figure 1.**
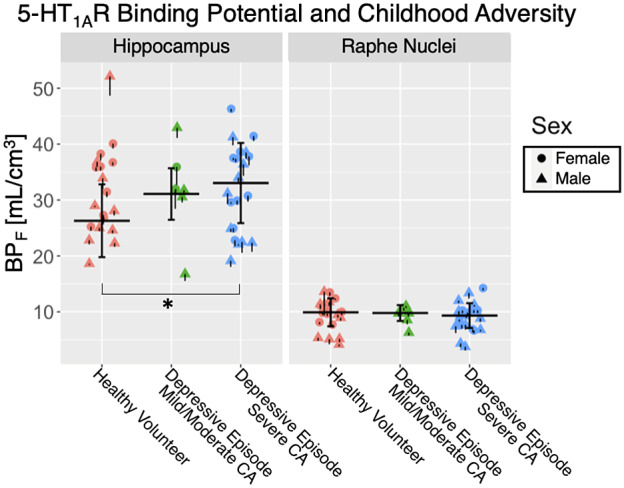
Scatter plots of 5-HT_1A_R BP_F_ values for the hippocampus and raphe nuclei for healthy volunteers (pink, *n* = 19), participants in a depressive episode with a reported history of mild-to-moderate childhood adversity (CA) (green, *n* = 7), and participants in a depressive episode with a reported history of severe CA (blue, *n* = 21). Females are shown with circles and males with triangles. The individual standard errors (SE) for each participant’s 5-HT_1A_R BP_F_ values are denoted by black vertical bars (positive SE bars omitted for clarity). Thick black bars show weighted group means and group SEs for each *a priori* region. A significant group-by-region interaction (*p* = 0.011) was driven by significantly higher hippocampal BP_F_ in participants in a depressive episode with severe CA compared to HVs (*p* = 0.019).

**Figure 2.**
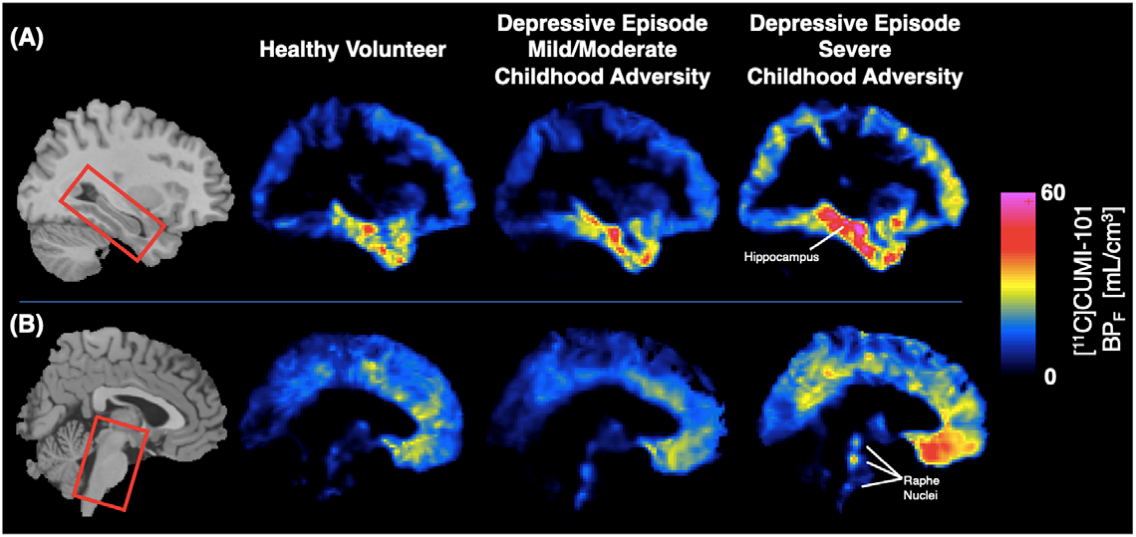
Representative [^11^C]CUMI-101 voxel maps of BP_F_ (mL/cm^3^) from each group, highlighting the hippocampus (A) and raphe nuclei (B). All images shown in Montreal Neurological Institute (MNI) space. Template MRI shown at left and a representative participant’s PET voxel map from each group shown in the right three columns.

The effect of CA on 5-HT_1A_R BP_F_ significantly differed by brain region (*F* = 5.08, *p* = 0.011). This was driven primarily by an elevation of 25.7%, on average, in hippocampal 5-HT_1A_R BP_F_ in participants with severe CA compared with HVs (*t* = 2.43, *p* = 0.019; Figures 1 and 2). There was no significant main effect of CA on 5-HT_1A_R BP_F_ across brain regions (*F* = 0.42, *p* = 0.66), and no differences were observed in BP_F_ between any pair of groups in the raphe nuclei, nor in the hippocampus between the mild/moderate adversity and severe adversity depressive episode groups (*p* *-value* *s* > 0.05). There was a significant main effect of sex (female > male, *F* = 4.10, *p* = 0.049), included as a control variable. There was a significant region-specific effect of HV versus all participants in a depressive episode (MDD and BD combined) on 5-HT_1A_R BP_F_ (*F* = 10.10, *p* = 0.003). This was driven by a significant elevation of 24.3%, on average, in hippocampal 5-HT_1A_R BP_F_ in participants in a depressive episode compared to HVs (*F* = 2.48, *p* = 0.017). Further analyses splitting participants in a depressive episode into MDD and BD groups are included in Supplementary Materials. This indicates an effect of depressive episode on hippocampal 5-HT_1A_R BP_F_, but also given the stepwise nature of highest 5-HT_1A_R BP_F_ in the participants in a depressive episode with severe CA, followed by mild/moderate CA, and then HV, it may indicate a dependence of 5-HT_1A_R BP_F_ on CA in depressive disorders.

### Exploratory analyses

For participants in a depressive episode, the continuous summed score across the CTQ subscales was not significantly associated with 5-HT_1A_R BP_F_ (*F* = 0.20, *p* = 0.66), nor was there a region-specific effect (*F* = 0.12, *p* = 0.73). When HVs were added, a significant continuous CTQ-by-region effect (*F* = 5.11, *p* = 0.03) was present; however, the correlation between CTQ summed over the five subscales and 5-HT_1A_R BP_F_ was not significant for either region alone (hippocampus: *B* = 0.001, *t* = 0.79, *p* = 0.43; raphe nuclei: *B* = -0.002, *t* = −1.17, *p* = 0.24).

To test for a potential sex difference in the severe CA versus HV effect, we added a three-way interaction of region-by-sex-by-CA in the original LME, which was not significant (*F* = 0.39, *p* = 0.68). Post-hoc tests confirmed this lack of sex effect with the severe CA versus HV effect in the hippocampus not being significantly different between males and females (*B* = 0.07, *t* = 0.52, *p* = 0.60).

Extending beyond the *a priori* hippocampal and raphe nuclei regions, we analyzed an additional 11 brain regions that comprise the full set of brain regions used in prior studies on the 5-HT_1A_R from our group [13, 31, 33, 40, 61, 62]. The effect of CA on 5-HT_1A_R BP_F_ again significantly differed by brain region (*F* = 1.97, *p* = 0.004), with the post-hoc significant effect of hippocampal 5-HT_1A_R BP_F_ in participants with severe CA versus HVs remaining and additionally a significant elevation of 17.0% in parahippocampal gyrus 5-HT_1A_R BP_F_ in participants with severe CA versus HVs (*t* = 0.22, *p* = 0.005).

An ANOVA found no evidence of group differences in hippocampal gray matter volume (*F* = 0.778, *p* = 0.67), and hippocampal gray matter volume was not correlated with SE-weighted BP_F_ values across groups (*r* = 0.05, *p* = 0.72). Further exploratory analyses on hippocampal subfields were performed and found no significant differences between groups (Supplementary Tables S3 and S4).

## Discussion

This is the first PET study, to our knowledge, to directly assess the relationship between reported CA and 5-HT_1A_R binding potential in depressive episodes using the weak partial agonist tracer, [^11^C]CUMI-101. Participants in a current depressive episode who reported severe CA had higher hippocampal 5-HT_1A_R BP_F_ than HVs. If this finding is replicated in a larger sample, it could be used to enhance development of novel strategies for treatment and prevention of depressive disorders in the context of CA. Our results, taken together with previous evidence of a strong relationship between CA and depression [2–4, 8, 9], suggest a common biological underpinning. Although assessment of causality was not possible given the cross-sectional nature of this study, our findings suggest that one possible mechanism by which CA might contribute to development and maintenance of depressive episodes in adulthood is via the serotonin system. Our results build on the hypothesis that stress early in life, a critical developmental period for 5-HT_1A_R expression, may contribute to lifelong serotonergic system aberrations, which may impact the onset and progression of mood disorders into adulthood [11, 12, 18, 21]. This hypothesis echoes the stress-diathesis model for depression which implicates the multiplicative effect of depression risk conferred by stress and genetic risk [63, 64].

CA is predictive of a more severe course of, and poorer clinical outcomes for, BD and MDD such as earlier age of onset, greater lifetime number of depressive episodes, treatment non-response, and increased risk for suicide [3, 4, 10]. Participants with BD or MDD who report CA may represent a distinct subgroup with different underlying neurobiology. For instance, Duarte et al. observed a negative correlation between severity of CA and gray matter volume in the prefrontal cortex and thalamus of patients with BD [65]. Further, adults with MDD who experienced CA presented with decreased gray matter volume in areas including the hippocampus [42], although no differences in hippocampal gray matter volume were found in the current study. Research suggests that the hippocampus may be especially sensitive to environmental stressors that occur during development [41]. CA appears to have an influence on hippocampal development, which may impact stress reactivity and causes a predisposition for the development of depressive symptomatology in adulthood [12, 15, 18, 43].

Contrary to our hypothesis, we did not observe a difference in raphe nuclei 5-HT_1A_R BP_F_ between groups based on CA history. The raphe nuclei are the sites of 5-HT synthesis and release [43, 44], and 5-HT_1A_ raphe nuclei autoreceptors regulate 5-HT neurotransmission throughout the brain by controlling serotonin neuron firing and release [66]. Evidence of 5-HT_1A_R involvement in CA response has been mixed in the hippocampus and raphe nuclei. In the hippocampus, acute and chronic corticosterone [67, 68] and acute stress [69] have been shown to reduce 5-HT_1A_R binding and mRNA levels in rodents, possibly through multiple transcriptional repression mechanisms involving both glucocorticoid and mineralocorticoid receptors [70, 71]. In the raphe nuclei, differences in 5-HT_1A_R function [72], 5-HT synthesis [73], and 5-HT_1A_R mRNA expression [74] were found in rodents with early life stress. Bravo et al. [75] found that maternal separation reduced 5-HT_1A_R mRNA expression in the raphe nuclei, with no change in the hippocampus, and Vázquez et al. [76] found the opposite, with maternal separation elevating 5-HT_1A_R mRNA expression in the hippocampus, with no change in the raphe nuclei. It is possible that after CA, during the transition to adulthood, there is a compensatory induction of 5-HT_1A_R that results in increased hippocampal 5-HT_1A_R, but a full-time course has yet to be reported.

There are a few factors that may account for our findings. First, there was an effect of sex on the results of those studies. Although our small sample size prevented us from fully being able to test interaction effects with sex, there was a main effect of sex in both of our statistical models. Future research could be done with a larger sample to test for sex-dependent effects of CA on 5-HT_1A_R binding potential in the raphe nuclei.

Second, prior studies did not look for dose-dependent effects of CA. While we found a significant difference in hippocampal 5-HT_1A_R between participants in a depressive episode reporting severe CA and HVs, visual inspection of group means also suggests a potential stepwise effect of reported CA on 5-HT_1A_R BP_F_ (Figure 2). It is possible that the small size of our mild/moderate CA group limited our ability to detect significance in post-hoc effects with the severe adversity and HV groups statistically, and further research is warranted. Differences in 5-HT_1A_ autoreceptor functioning in the raphe nuclei may represent a prepotent vulnerability that, in the context of CA, can lead to depression. Evidence suggests that there is a critical developmental period during which early stress can lead to long-term changes in 5-HT_1A_R density in projection targets such as the hippocampus [21]. Further, these changes appear to result in reduced plasticity of the serotonergic system that, when combined with stress in adulthood, could result in stress-related psychiatric morbidities such as depression [74]. More research on the interaction between CA and 5-HT_1A_ in the raphe nuclei is needed to understand how early life experiences contribute to the development of depressive disorders.

Finally, [^11^C]-WAY100635 is a 5-HT_1A_R antagonist and as such binds 5-HT_1A_Rs independent of whether they are in the high or low affinity state, whereas the currently used [^11^C]-CUMI-101 has been shown to be a partial agonist in [^35^S]GTPgS binding studies in human 5-HT_1A_-transfected Chinese Hamster Ovary cells [77], but also has functional antagonist activity in cross-species examination [78]. Differences between [^11^C]-WAY100635 and [^11^C]-CUMI-101 conformational binding preference may explain the low binding in the raphe nuclei with [^11^C]-CUMI-101 [34, 45] relative to [^11^C]-WAY100635 [31, 61, 62], which could contribute to why we did not observe an effect of depression on 5-HT_1A_R binding in the raphe nuclei, but did in the hippocampus. The raphe nuclei are also populated with autoreceptors and the hippocampus heteroreceptors, with documented differences in 5-HT_1A_R signaling between the regions [79], which could also account for the disparate findings.

While studies using [^11^C]-WAY100635 have consistently found depressed versus HV differences in raphe nuclei 5-HT_1A_R [31, 33, 37], the [^11^C]CUMI-101-measured difference in 5-HT_1A_R binding here seems to be limited to the hippocampus. Two recent analyses with [^11^C]-CUMI-101 found differences in 5-HT_1A_R binding potential between BD and HV groups in the raphe nuclei and across a large set of regions of interest (ROIs) that included the hippocampus [34] and between a mixed-mood disorder sample at high risk for mood disorder comprising participants with MDD, BD, dysthymia, and depressive disorder not otherwise specified and HVs in the ventromedial prefrontal cortex and the medial orbitofrontal cortex [80]. However, in Ananth et al., there were no differences between BD and HV groups in the raphe nuclei or in a large set of ROIs [40]. The current study used the participants from Ananth et al. [40] with BD who completed the CTQ. We found here hippocampal differences between a combined BD + MDD group and HVs; however, we also found that when BD and MDD groups were compared in separate models to HVs, this difference did not persist (Supplementary Materials). We theorize that the discordance with Ananth et al. [40] may be due to increased power here to detect an effect given the combined BD + MDD sample relative to the sole BD sample in Ananth et al. [40], along with differences within the HV group. Studies with larger samples could test this theory. However, it is worth noting that the two regions of significant elevation in participants in a depressive episode with severe CA relative to HV, the hippocampus (*a priori*) and parahippocampus (exploratory), were two of the three brain regions in Ananth et al. [40] whose 5-HT_1A_R BP_F_ predicted lithium monotherapy treatment response. It may be that these two regions are particularly sensitive to effects that both contribute to the pathogenesis and ameliorate the progression of depressive disorders.

There are limitations to this study. First, our modest sample size included a set of HVs imaged at alternate sites (BNL *n* = 4 and CUIMC *n* = 1). However, we repeated our analyses and our findings persisted when only including those scanned at Yale (See Supplementary Materials). Although we did control for the effect of sex on 5-HT_1A_R binding potential (female > male), it is possible that the mild/moderate CA group’s lower 5-HT_1A_R binding potential than the severe CA group was driven by the mild/moderate CA group not being well matched for sex, with only two of the seven participants being female. Further, there have been mixed findings regarding age effects on 5-HT_1A_R with some studies reporting no association [29] and others showing 5-HT_1A_R binding potential decreases with age, particularly in females, in humans [81, 82] and nonhuman primates [83]. Although age effects in this study did not reach statistical significance, it is possible that the limited sample size and age range inhibited us from detecting an effect.

Second, the CTQ is a retrospective self-report questionnaire and subject to recall bias, especially in participants with depression. However, it is a sensitive and valid screening tool for assessment of CA in psychiatric settings [84]. Participants were grouped by severity of CA based on the CTQ manual [49]. This method classifies individuals reporting severe abuse or neglect in at least one CTQ domain as having experienced severe adversity but does not account for a cumulative effect of adversity across multiple domains. Although scores are influenced by several dimensions of CA [49], the CTQ is unable to capture on a true scale the severity, duration, and subjective experience of adversity [49]. We did perform exploratory analyses on the summed scores across all CTQ domains, treating it as a continuous measure; however, the stepwise increase in 5-HT_1A_R BP_F_ from HVs to participants in a depressive episode with severe CA was not reflected in the continuous analyses, with no significant correlations between summed CTQ and 5-HT_1A_R BP_F_ at the regional level.

Third, all but one participant in a depressive episode reported some degree of childhood trauma. Therefore, it was not feasible to assess 5-HT_1A_R BP_F_ in participants in a depressive episode without CA. However, mild/moderate and severe CA subgroups did not differ on any other symptom measure, suggesting that our results are not confounded by clinical variables such as depression, anxiety, or suicidality. Furthermore, the high frequency of CA in our depressive episode sample is consistent with the marked relationships between early adversity and BD and MDD that have been reported in the literature [4]. However, the lack of continuous correlation between summed CTQ and 5-HT_1A_R BP_F_, the limited size of the mild/moderate CA group, and the fact that all participants in a depressive episode reported CA all combine to limit our ability to disentangle the depression effect from the CA effect. Future studies with larger samples of participants in a depressive episode should seek to clarify these effects.

Lastly, given the cross-sectional nature of our investigation, assessment of CA as a causal factor in the development of BD or MDD was beyond the scope of this study. However, these initial results, consistent with previous findings of a relationship between BD or MDD and CA, and in conjunction with the congruent animal studies designed to assess causality described above, support this interpretation.

In summary, this is the first investigation of the relationship between CA and 5-HT_1A_R binding potential using [^11^C]CUMI-101 PET in currently depressed participants with BD or MDD compared to HVs. The primary finding was that participants in a depressive episode with severe CA had higher hippocampal 5-HT_1A_R BP_F_ than HVs. These results suggest an interplay between CA, depressive symptomatology in adulthood, and alterations of 5-HT_1A_R in the hippocampus. Follow-up in larger samples, especially given the small sample size of the mild/moderate CA group, and attempts to assess causality in the link between CA, depression, and the 5-HT_1A_R are necessary. A central 5-HT_1A_R BP_F_ biomarker of depression comorbid with CA may have the potential to be applied toward the future development of interventions for prevention and treatment of depressive disorders.

## Supplementary material

For supplementary material accompanying this paper visit http://doi.org/10.1192/j.eurpsy.2023.4. click here to view supplementary material
