# Supplementary material for: In vivo serotonin 1A receptor hippocampal binding potential in depression and reported childhood adversity
Source: Eur Psychiatry. 2023 Jan 24;66(1):e17. doi: 10.1192/j.eurpsy.2023.4 (PMC9970152; doi:10.1192/j.eurpsy.2023.4)
Supplement: Supplementary file 1 [file S0924933823000044sup001.docx]

**Supplemental Information**

**Methods**

Due to the unbalanced nature of the cohorts, site could not be added as an effect in the primary analysis models. To ensure our results were not driven by differences in site, we repeated our analyses using only those participants that were scanned at Yale University. A linear mixed-effects model was used to compare serotonin 1A receptor (5-HT_1A_R) binding potential (BP_F_) in the *a priori* regions of interest (ROIs) (raphe nuclei and hippocampus) between participants in a depressive episode (n=28) and the subset of healthy volunteers (HVs) (n=14) who underwent their [^11^C]CUMI-101 PET scans at Yale University. This analysis was identical to that of the primary analysis with the entire cohort, but excluded those HVs that were scanned at BNL (n=4) and CUIMC (n=1). Specifically, first the three-group model (depressive episode and severe childhood adversity, depressive episode and mild/moderate childhood adversity, and HVs) was run, followed by the two-group (participants in a depressive episode vs. HVs) model. In addition to group, sex and ROI were also fixed effects, and participant was a random effect.

To ensure any effect of diagnostic group (participants in a depressive episode vs. HVs) was not due to differences between participants with bipolar depression (BD) and major depressive disorder (MDD), a linear mixed effects model was also fit using diagnosis (MDD vs BD) (with sex and ROI as fixed effects and participant as a random effect). We also further fit models with diagnosis as MDD vs HV and BD vs HV separately to separate any MDD/BD effects.

In all models, within and between participant covariance were modeled using unstructured variance and random intercepts. Models showed good fit to the data as indicated by the Akaike Information Criterion.

**Results**

Consistent with the primary analyses in the full sample, a significant group (participants in a depressive episode with severe childhood adversity, participants in a depressive episode with mild/moderate adversity, and HVs) by region interaction in 5-HT_1A_R BP_F_ was observed (F=4.64, *p*=0.016; Fig S1.). This effect was driven by significantly higher hippocampal 5-HT_1A_R BP_F_ in participants in a depressive episode reporting severe childhood adversity compared to HVs (t=2.38, *p*=0.022; Fig S2.). Hippocampal 5-HT_1A_R BP_F_ was higher in participants with mild/moderate adversity compared to HVs, following the stepwise nature of the 5-HT_1A_R binding potential effect reported in the manuscript, but again this difference did not reach statistical significance (t=0.98, *p*=0.34; Fig S2). Similarly, there was no significant difference in hippocampal 5-HT_1A_R BP_F_ between severe and mild/moderate childhood adversity groups (t=0.50, *p*=0.62; Fig S2). In the raphe nuclei, no differences in 5-HT_1A_R BP_F_ were observed across groups (*p*=0.38 to 0.56; Fig S2). Also, a significant diagnostic group (participants in a depressive episode *vs* HVs) by region effect was found in 5-HT_1A_R BP_F_ across the *a priori* ROIs (F=9.21, *p*=0.004). This effect was driven primarily by a significant region-specific elevation of hippocampal 5-HT_1A_R BP_F_ in participants in a depressive episode relative to HVs (t= 2.40, *p*=0.02). In the raphe nuclei, no significant difference in 5-HT_1A_R BP_F_ was observed between groups (t= 0.94, *p*=0.35).

When the depressive episode sample was compared across BD and MDD diagnoses, there was no significant main effect of diagnosis (F = 0.03, *p* = 0.87), and no diagnosis-by-region interaction (F = 1.58, *p* = 0.22). This was also the case across MDD and HV diagnoses (main effect: F=0.11, p=0.75; diagnosis-by-region interaction: F=1.45, p=0.24). Across BD and HV diagnoses, similarly, there was no significant main effect of diagnosis (F=1.27, p=0.27). While there was a significant diagnosis-by-region interaction (F=6.47, p=0.02), neither regions individually had significant BD vs HV differences (p=0.09 for hippocampus, p=0.39 for raphe nuclei).

**Supplemental Figures**

***
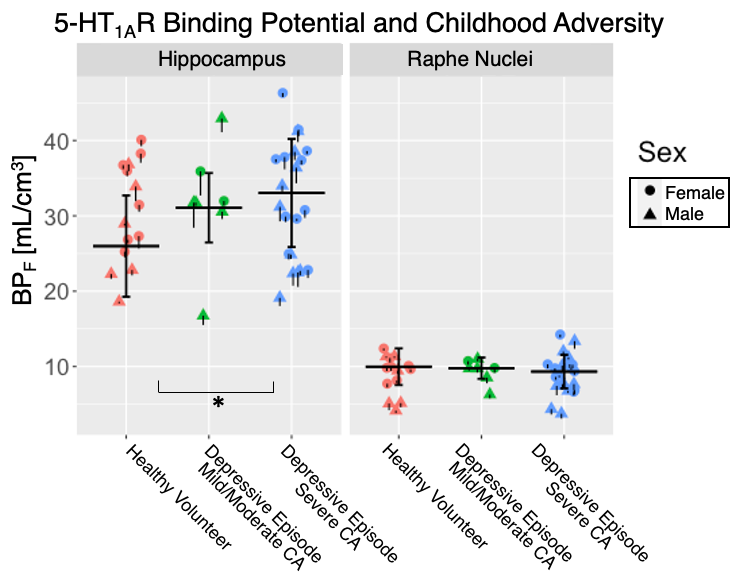
***

***Figure S1.*** Scatter plots of 5-HT_1A_R BP_F_ (mL/cm^3^) values for the hippocampus and raphe nuclei for healthy volunteers scanned at Yale University (white, n=14), participants in a depressive episode with a reported history of mild/moderate childhood adversity (CA) (pink, n=7), and participants in a depressive episode with a reported history of severe CA (red, n=21). The individual standard errors (SE) for each participant’s 5-HT_1A_R BP_F_ values are denoted by black vertical bars (positive SE bars omitted for clarity). Thick black bars show weighted group means and group SEs for each *a priori* region.

**Supplemental Tables**

***Table S1.*** Cut-off scores for the Childhood Trauma Questionnaire (CTQ), by subscale. Patients with a score above the cut-off for “severe” on one-or-more subscale were categorized into the severe childhood adversity group.

| Subscale | None | Low | Moderate | Severe |
| --- | --- | --- | --- | --- |
| Emotional Abuse | 5-8 | 9-12 | 13-15 | 16+ |
| Physical Abuse | 5-7 | 8-9 | 10-12 | 13+ |
| Sexual Abuse | 5 | 6-7 | 8-12 | 13+ |
| Emotional Neglect | 5-9 | 10-14 | 15-17 | 18+ |
| Physical Neglect | 5-7 | 8-9 | 10-12 | 13+ |

***Table S2***. Summarizes ANOVA group comparisons of [^11^C]CUMI-101 radiochemistry.

|  | Healthy Volunteers (n=19)  mean(SD) | Participants in a Depressive Episode with Mild/Moderate Childhood Adversity  (n=7)  mean(SD) | Participants in a Depressive Episode with Severe Childhood Adversity  (n=21)  mean(SD) |  |
| --- | --- | --- | --- | --- |
| Injected Dose (mCi) | 14.6(5.20) | 13.0(4.15) | 16.4(3.66) | F(2,44) = 1.855, *p* = 0.17 |
| Injected Mass (µg) | 1.82(1.35) | 1.24(0.81) | 1.06(0.60) | F(2,43) = 2.88, *p* = 0.07 |
| Plasma Free Fraction (f_P_) | 0.41(0.07) | 0.41(0.08) | 0.37(0.05) | F(2,44) = 2.40, *p* = 0.10 |
| Specific Activity (mCi/nmol) | 5.45(3.93) | 4.92(2.59) | 7.21(3.13) | F(2,43) = 1.86, *p* = 0.17 |

***Table S3.*** Summarizes the results of exploratory two-tailed, two-sample Welch’s *t*-test analyses comparing gray matter volumes of hippocampal subregions between groups (healthy volunteers (HV), participants in a depressive episode with mild-to-moderate childhood adversity, participants in a depressive episode with severe childhood adversity).

| Region | Group 1 | Group 2 | *df* | *t* | *p* |
| --- | --- | --- | --- | --- | --- |
| left_CA1 | HV | Mild/Moderate | 8.02 | 0.58 | 0.58 |
|  | HV | Severe | 33.12 | 0.86 | 0.39 |
|  | Mild/Moderate | Severe | 10.49 | 0.01 | 0.99 |
| left_CA2_3 | HV | Mild/Moderate | 9.04 | 1.32 | 0.22 |
|  | HV | Severe | 35.82 | 1.32 | 0.20 |
|  | Mild/Moderate | Severe | 10.73 | 0.38 | 0.71 |
| left_CA4_DG | HV | Mild/Moderate | 10.14 | 1.13 | 0.29 |
|  | HV | Severe | 36.6 | 1.05 | 0.30 |
|  | Mild/Moderate | Severe | 11.46 | 0.33 | 0.75 |
| left_fimbria | HV | Mild/Moderate | 17.0 | 0.30 | 0.76 |
|  | HV | Severe | 29.32 | 0.36 | 0.72 |
|  | Mild/Moderate | Severe | 10.0 | 0.71 | 0.50 |
| left_hippocampal_fissure | HV | Mild/Moderate | 12.17 | 1.44 | 0.18 |
|  | HV | Severe | 35.29 | 0.10 | 0.92 |
|  | Mild/Moderate | Severe | 10.17 | 1.44 | 0.18 |
| left_presubiculum | HV | Mild/Moderate | 13.60 | 0.33 | 0.45 |
|  | HV | Severe | 35.32 | 0.19 | 0.85 |
|  | Mild/Moderate | Severe | 11.21 | 0.19 | 0.85 |
| left_subiculum | HV | Mild/Moderate | 7.62 | 0.16 | 0.87 |
|  | HV | Severe | 36.38 | 0.39 | 0.70 |
|  | Mild/Moderate | Severe | 8.25 | 0.04 | 0.97 |
| Left-Hippocampus | HV | Mild/Moderate | 7.71 | 0.18 | 0.86 |
|  | HV | Severe | 36.44 | 1.05 | 0.30 |
|  | Mild/Moderate | Severe | 8.35 | 0.38 | 0.71 |
| right_CA1 | HV | Mild/Moderate | 8.80 | 0.56 | 0.59 |
|  | HV | Severe | 36.0 | 0.46 | 0.65 |
|  | Mild/Moderate | Severe | 10.23 | 0.24 | 0.81 |
| right_CA2_3 | HV | Mild/Moderate | 8.16 | 0.64 | 0.54 |
|  | HV | Severe | 36.61 | 1.53 | 0.13 |
|  | Mild/Moderate | Severe | 8.84 | 0.24 | 0.81 |
| right_CA4_DG | HV | Mild/Moderate | 8.15 | 0.46 | 0.66 |
|  | HV | Severe | 37.0 | 1.13 | 0.27 |
|  | Mild/Moderate | Severe | 8.33 | 0.17 | 0.87 |
| right_fimbria | HV | Mild/Moderate | 12.16 | 0.03 | 0.98 |
|  | HV | Severe | 36.88 | 0.42 | 0.68 |
|  | Mild/Moderate | Severe | 11.84 | 0.36 | 0.72 |
| right_hippocampal_fissure | HV | Mild/Moderate | 11.76 | 0.03 | 0.97 |
|  | HV | Severe | 32.96 | 0.22 | 0.83 |
|  | Mild/Moderate | Severe | 8.90 | 0.13 | 0.90 |
| right_presubiculum | HV | Mild/Moderate | 8.80 | 0.99 | 0.35 |
|  | HV | Severe | 35.58 | 0.25 | 0.81 |
|  | Mild/Moderate | Severe | 7.95 | 0.88 | 0.40 |
| right_subiculum | HV | Mild/Moderate | 7.78 | 0.77 | 0.47 |
|  | HV | Severe | 36.38 | 0.83 | 0.41 |
|  | Mild/Moderate | Severe | 8.48 | 0.31 | 0.76 |
| Right-Hippocampus | HV | Mild/Moderate | 9.44 | 0.25 | 0.81 |
|  | HV | Severe | 36.83 | 1.34 | 0.19 |
|  | Mild/Moderate | Severe | 9.17 | 0.61 | 0.56 |
| Total-Hippocampus | HV | Mild/Moderate | 8.27 | 0.23 | 0.83 |
|  | HV | Severe | 36.87 | 1.30 | 0.20 |
|  | Mild/Moderate | Severe | 8.72 | 0.52 | 0.62 |

***Table S4***. Summarizes the results of exploratory one-tailed two-sample Welch’s *t*-test analyses comparing gray matter volumes of hippocampal subregions between participants in a depressive episode and healthy volunteers

| Region | *df* | *t* | *p* |
| --- | --- | --- | --- |
| left_CA1 | 43.96 | 0.96 | 0.34 |
| left_CA2_3 | 43.10 | 1.61 | 0.11 |
| left_CA4_DG | 41.58 | 1.29 | 0.20 |
| left_fimbria | 27.31 | 0.17 | 0.87 |
| left_hippocampal_fissure | 35.38 | 0.60 | 0.55 |
| left_presubiculum | 33.73 | 0.27 | 0.79 |
| left_subiculum | 43.64 | 0.39 | 0.70 |
| Left-Hippocampus | 43.55 | 0.90 | 0.37 |
| right_CA1 | 43.07 | 0.61 | 0.55 |
| right_CA2_3 | 42.92 | 1.50 | 0.14 |
| right_CA4_DG | 41.98 | 1.09 | 0.28 |
| right_fimbria | 37.68 | 0.33 | 0.75 |
| right_hippocampal_fissure | 32.10 | 0.18 | 0.86 |
| right_presubiculum | 38.53 | 0.65 | 0.52 |
| right_subiculum | 43.52 | 1.02 | 0.31 |
| Right-Hippocampus | 39.41 | 1.15 | 0.26 |
| Total-Hippocampus | 42.47 | 1.12 | 0.27 |
